# The First Illumina-Based *De Novo* Transcriptome Sequencing and Analysis of Safflower Flowers

**DOI:** 10.1371/journal.pone.0038653

**Published:** 2012-06-19

**Authors:** Huang Lulin, Yang Xiao, Sun Pei, Tong Wen, Hu Shangqin

**Affiliations:** Department of Traditional Chinese Medicine Study, Industrial Crop Institute, Sichuan Academy of Agricultural Sciences, Jianyang, Sichuan, China; Auburn University, United States of America

## Abstract

**Background:**

The safflower, *Carthamus tinctorius* L., is a worldwide oil crop, and its flowers, which have a high flavonoid content, are an important medicinal resource against cardiovascular disease in traditional medicine. Because the safflower has a large and complex genome, the development of its genomic resources has been delayed. Second-generation Illumina sequencing is now an efficient route for generating an enormous volume of sequences that can represent a large number of genes and their expression levels.

**Methodology/Principal Findings:**

To investigate the genes and pathways that might control flavonoids and other secondary metabolites in the safflower, we used Illumina sequencing to perform a *de novo* assembly of the safflower tubular flower tissue transcriptome. We obtained a total of 4.69 Gb in clean nucleotides comprising 52,119,104 clean sequencing reads, 195,320 contigs, and 120,778 unigenes. Based on similarity searches with known proteins, we annotated 70,342 of the unigenes (about 58% of the identified unigenes) with cut-off E-values of 10^−5^. In total, 21,943 of the safflower unigenes were found to have COG classifications, and BLAST2GO assigned 26,332 of the unigenes to 1,754 GO term annotations. In addition, we assigned 30,203 of the unigenes to 121 KEGG pathways. When we focused on genes identified as contributing to flavonoid biosynthesis and the biosynthesis of unsaturated fatty acids, which are important pathways that control flower and seed quality, respectively, we found that these genes were fairly well conserved in the safflower genome compared to those of other plants.

**Conclusions/Significance:**

Our study provides abundant genomic data for *Carthamus tinctorius* L. and offers comprehensive sequence resources for studying the safflower. We believe that these transcriptome datasets will serve as an important public information platform to accelerate studies of the safflower genome, and may help us define the mechanisms of flower tissue-specific and secondary metabolism in this non-model plant.

## Introduction

The safflower, *Carthamus tinctorius* L., is a member of the Compositae or Asteraceae plant family, and is cultivated mainly for its seeds and flowers. The safflower plant yields a variety of products, including the numerous secondary metabolites of its flowers and the unsaturated fatty acids in its seeds, many of which are very beneficial to human health. Although the safflower is a minor crop, it is one of the oldest crops in human history, and it is cultivated worldwide; in 2007, safflower cultivation accounted for ∼ 795,118 ha across the globe [1]. Traditionally, this crop was grown for its flowers, which were a source of yellow and red dyes for clothing and food. Safflower oil first was first used in the paint industry, but due to its health benefits, it is now produced commercially for cooking oil and other uses [2], [3], [4], [5].

In plants, primary metabolites are organic compounds that are directly involved in normal growth, development and reproduction. In contrast, secondary metabolites often play important roles in defending the plant against herbivores and diseases. The flavonoids are a large group of secondary metabolites that humans have long used for medicines [6], [7], toning, and other purposes. Safflower flavonoid extracts have been clinically used for the prevention and treatment of cardiovascular disease in China [6], [8] and Japan for centuries. The safflower is a native plant whose flowers have long been processed into a Chinese herbal medicine called *Chuanhonghua* in China Jianyang country. In China, safflower is mainly cultivated for the medicinal properties of its flower tissues. To date, ∼ 50 secondary metabolites have been identified in safflower flower extracts; most of them are generalized flavonoids, a class of modulators that mediate bifunctional interactions at vicinal ATP- and steroid-binding sites [9]. These include carthamone, safflor yellow A, hydroxysafflor yellow A, 6-hydroxykaempferol, 6-hydroxykaempferol-3-o-β-D-glucoside, kaempferol [10], [11], [12], and others. The abundance of flavonoids as secondary metabolites makes the safflower a good model for investigating flavonoid biosynthesis in plants, and the related genes and pathways. This should provide a better understanding of the genes responsible for regulating natural products, and could help us improve the use of these natural products for human health.

In recent years, researchers have focused on examining the genetic diversity of the safflower. For example, Pahlavani *et al*. reported on the inheritance of flower color and spininess [13], while Chapman *et al*. reported on the DNA sequence diversity and origins of the cultivated safflower, along with its development and polymorphisms, and the cross-taxon utility of EST-SSR markers [14], [15], [16]. More recently, Li *et al*. found that there are at least 236 known microRNAs (miRNAs) expressed in the safflower, 100 of which are conserved across plants [17]. To date, however, the safflower genome has not been fully sequenced, and relatively little is known about its encoded genes. As of October 2011, only 567 nucleotide sequences, 41,588 expressed sequence tags (ESTs), 162 proteins and 0 genes from *Carthamus tinctorius* had been deposited in the NCBI’s GenBank database.

EST sequencing has traditionally been the core technology used for the discovery of reference transcripts. However, it has some inherent limitations, such as low throughput, high cost and a long experimental cycle. In recent years, researchers have developed a high-throughput sequencing technology called Next Generation Sequencing (NGS) [18]. Various platforms utilize NGS, such as the Illumina Genome Analyzer, the Roche/454 Genome Sequencer FLX Instrument, and the ABI SOLiD System; these have proven to be powerful and cost-effective tools for advanced research in many areas, including genome sequencing, genome re-sequencing, miRNA expression profiling, DNA methylation analysis, and especially the *de novo* transcriptome sequencing of non-model organisms [19], [20]. This method of transcriptome analysis is fast and simple because it does not require bacterial cloning of the cDNAs. Instead, direct cDNA sequencing generates an extraordinary depth of short reads. It is a more comprehensive and efficient way to measure transcriptome composition, obtain RNA expression patterns, and discover new genes. In addition, this approach is very sensitive, and thus allows the detection of low-abundance transcripts. Recent transcriptomic studies on *Arabidopsis thaliana* [21], mouse [22], and human [23] cells have demonstrated that this approach is well-suited for surveying the complexity of eukaryotic transcriptomes by *de novo* assembly.

In this study, we used NGS technology to survey the poly (A) + transcriptome of *Carthamus tinctorius* L. flower tissues. The coverage of the transcriptome was ∼ 4.7 Gb of clean nucleotides, and thus comprehensive enough to discover all known genes and major metabolic pathways in this transcriptome. This dataset will serve as a public information platform for gene expression, genomics, and functional genomics in *Carthamus tinctorius* L. The assembled and annotated transcriptome sequences provide a valuable resource for identifying most of the genes expressed in the safflower.

## Results

### Flower Transcriptome Sequencing Output and *de novo* Assembly

**Figure 1:**
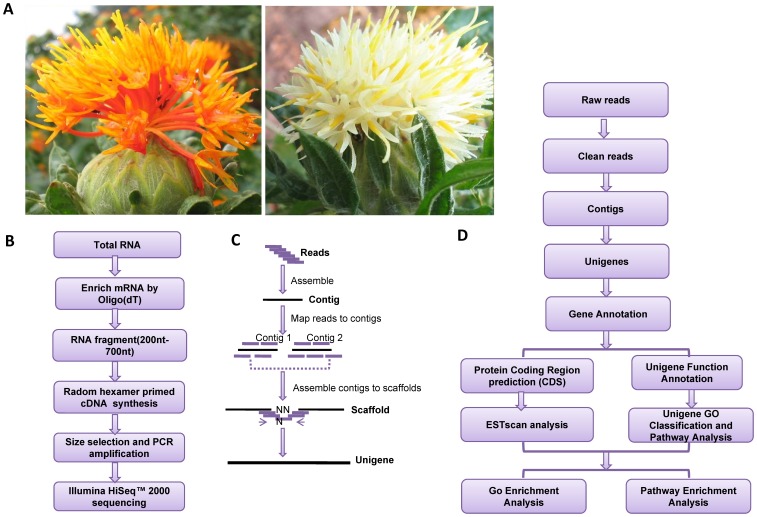
Safflower flower phenotypes and schematics of the transcriptome sequencing analysis. (A) Phenotypes of the red/orange (left) and white (mutant; right) flowers of *Carthamus tinctorius* L. used in this study. (B) Overall workflow of the experiment. (C) Overall workflow of the data assembly. (D) Overall workflow of the bioinformatic analysis.

To comprehensively cover the safflower flower transcriptome, total RNA was extracted from the tubular tissues of red/orange and white flowers. The phenotypes of each color are shown in Figure 1A. Flowers were collected on the 1st, 2nd and 3rd days of the flowering stage. Equal amounts of total RNA from each sample were pooled together, and mRNA was isolated, enriched, sheared into smaller fragments, and reverse-transcribed into cDNA. The cDNA were subjected to Illumina HiSeq™ 2000 sequencing, and the resulting sequencing data were subjected to bioinformatic analysis (Figure 1B–D). After removal of adaptor sequences, ambiguous reads and low-quality reads (Q20<20), we obtained a total of 52,119,104 clean reads of 75-bp long comprising 4,690,719,360 nucleotides (4.69 Gb) of high-quality clean reads. An overview of the sequencing and assembly is outlined in Table 1. All high-quality reads were assembled *de novo* using the Trinity program [24], which produced 195,230 contigs, with an N50 of 290 bp (i.e. 50% of the assembled bases were incorporated into contigs of 290 bp or longer).

**Table 1:** Summary of the sequence assembly after Illumina sequencing.

	Sequences (nt)	All numbers	Mean length (bp)	N50 (bp)
Total Clean reads	4,690,719,360	52,119,104	75	
Total Contigs	56,526,416	195,230	255	290
Total unigenes	53,897,307	120,778	446	555
GC percentage				46.97%
N percentage				0.00%
Q20 percentage				90.73%

**Figure 2:**
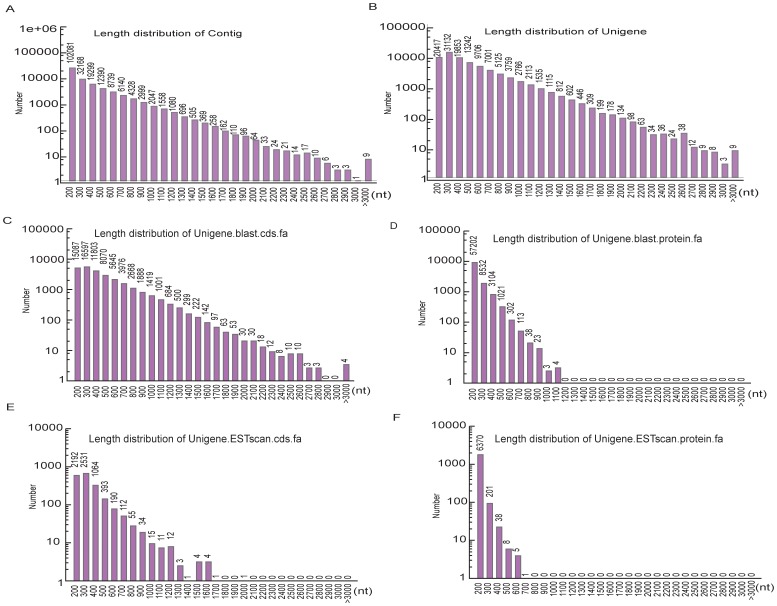
Overview of the safflower flower tissue transcriptome assembly. (A) The size distribution of the contigs obtained from our *de novo* assembly of high-quality clean reads. (B) The size distribution of the unigenes produced from further assembly of contigs (i.e., contig joining, gap filling, and scaffold clustering). (C) The size distribution of the CDS produced by searching unigene sequences against various protein databases (Nr, SwissProt, KEGG and COG, in order) using BLASTX (E-value <10^−5^). (D) The size distribution of the proteins predicted from the CDS sequences. (E) And (F) Size distributions of the ESTs and proteins obtained from the ESTScan results. For unigene CDS that had no hits in the databases (Nr, SwissProt, KEGG and COG), the BLAST results were subjected to ESTScans and then translated into peptide sequences.

The size distribution of the safflower contigs is shown in Figure 2A. A total of 195,230 contigs were assembled; the mean length was 255 bp, and although the majority of the contigs were less than 200 bp, 41,682 of the contigs were greater than 500 bp in length. A total of 120,778 unigenes were assembled, with an average unigene length of 446 bp and an N50 of 555 bp (the size distribution is shown in Figure 2B). Of the 120,778 unigenes, 49,376 were longer than 500 bp; 10,543 were longer than 1,000 bp; and 1,600 were longer than 1,500 bp. Although the unigene distribution closely followed the contig distribution, with the majority being shorter sequences, we obtained much longer unigenes compared to contigs (Figure 2A and 2B). The longest unigene recovered in this work was Unigene1325_safflower, which is 3,967 bp and encodes a splicing factor 3B subunit 1 (according to SwissProt annotation).

For our analyses of coding sequences (CDS), predicted proteins, and gene annotations, all assembled sequences were first searched against the plant proteins found in the NCBI non-redundant database (Nr), and then against the Swiss-Prot, Kyoto Encyclopedia of Genes and Genomes pathway (KEGG) and Orthologous Groups of proteins (COG) [25] databases, using the BLASTX program (E-value <10^−5^). We obtained a total of 70,342 significant BLAST hits (58.24% of all unigenes). The size distribution for the CDS and predicted proteins are shown in Figure 2C and 2D. The CDS of the unigenes that did not have BLAST hits were predicted using ESTScan and then translated into peptide sequences [26]; 6,623 unigenes were analyzed using this method (the size distributions of the ESTs and proteins are shown in Figure 2E and 2F).

### Functional Annotation

Unigene annotations provide functional information, including protein sequence similarities, COG clusters, gene ontology (GO) and KEGG pathway information. We searched the safflower unigene sequences against the protein databases (Nr, SwissProt, KEGG and COG) using BLASTX (E-value <10^−5^) and predicted the protein functions from the annotations of the most similar proteins. Distinct gene sequences were first searched using BLASTX against the Nr database, and 67,980 unigenes (56.3% of all unigenes) had hits that exceeded the E-value cutoff. Similarly, 49,260 unigenes (40.8%) were identified from the SwissProt database. A total of 70,342 unigenes were annotated in one or more of the databases (about 58% of all assembled unigenes), suggesting they have relatively well conserved functions.

**Figure 3:**
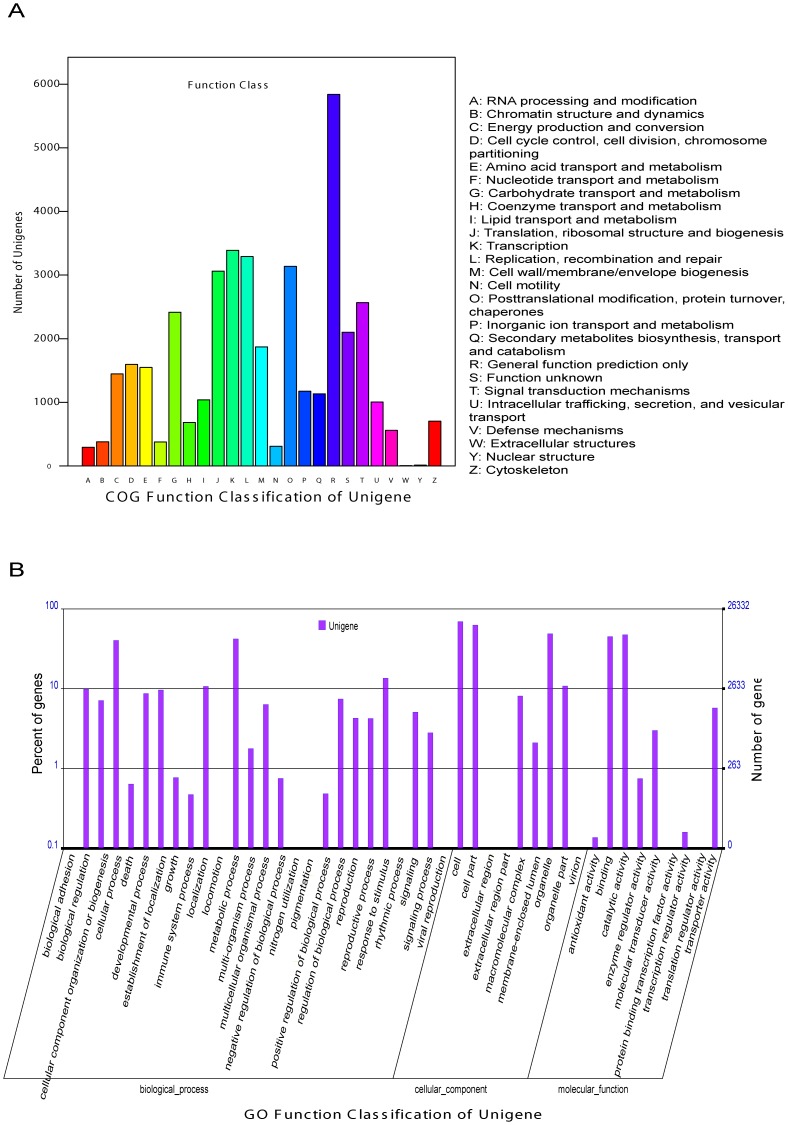
COG and GO classification of the safflower flower tissue transcriptome. (A) COG functional classification of the transcriptome. A total of 21,943 unigenes showed significant homologies to genes in the COG Nr database (E-value <1.0^−5^), distributing to 25 COG categories. (B) GO classification of the safflower flower tissue transcriptome. A total of 26,332 safflower unigenes were assigned to 1,754 GO term annotations using BLAST2GO, and then summarized into the three main GO categories and 44 sub-categories (functional groups).

The COG database contains classifications of orthologous gene products. Every protein in the COG is assumed to be evolved from an ancestor protein, and the database includes the coding proteins of complete genomes and the evolutionary relationships of bacteria, algae and eukaryotes. The safflower unigenes were searched against the COG database in order to predict and classify their possible functions. Out of 67,980 Nr hits, 21,943 sequences had COG classifications, distributing to 25 COG categories (Figure 3A). Among the 25 COG categories, “general function prediction only” represented the largest group (5,839; 26.6%), followed by “transcription” (3,389; 15.4%), “replication, recombination and repair” (3,291; 15.0%), “posttranslational modification, protein turnover, chaperones” (3,137; 14.3%) and “translation, ribosomal structure and biogenesis” (3,062; 14.0%). The smallest groups were “nuclear structures” (12 unigenes) and “extracellular structures” (5 unigenes) (Figure 3A).

GO is an international standardized gene-function classification system that uses a dynamically updated, controlled vocabulary and a strictly defined concept to comprehensively describe the properties of genes and their products in any organism. The GO database comprises three ontologies: molecular function, cellular components and biological processes. The basic units of GO are the “GO terms,” which each belong to a type of ontology. Here, we obtained the GO functional annotations of the safflower unigenes with Nr annotations by BLAST2GO program [27], and then used the WEGO software [28] to perform GO functional classifications for all of the unigenes and to examine the macro-level distribution of gene functions for this species. A total of 26,332 safflower unigenes were assigned 1,754 GO-term annotations using BLAST2GO, and the terms were summarized into the three main GO categories and 44 sub-categories (functional groups) (Figure 3B). In each of the three main categories of the GO classification (biological processes, cellular components and molecular functions), the dominant terms were “cellular processes,” “metabolic processes,” “cells,” “cell parts,” “organelles,” “binding,” and “catalytic activity.” More than half of the genes fell within the “biological processes” category. A high-percentage of the genes in the biological processes category fell under “cellular processes” and “metabolic processes;” a high percentage of the genes in the cellular components category fell under “cells,” “cell parts,” and “organelles”; and “binding” and “catalytic activity” dominated in the molecular function category (Figure 3B).

Pathway-based analysis can help us further understand the biological functions of genes. The KEGG pathway database contains information on networks of intracellular molecular interactions, and their organism-specific variations [29]. To identify the biological pathways in the safflower, we mapped the annotated sequences to the reference canonical pathways contained in the KEGG database. In total, we assigned 30,203 unigenes to 121 KEGG pathways (File S1). The most highly represented category was “metabolic pathways,” with 6,948 members. The “biosynthesis of secondary metabolites” and “plant-pathogen interaction” pathways were also well represented, with 3,598 members and 2,348 members, respectively. Many genes corresponded to pathways involved in the biosynthesis of secondary metabolites, including “flavonoid biosynthesis” (274 genes), “flavone and flavonol biosynthesis” (114 genes), “isoquinoline alkaloid biosynthesis” (77 genes), “tropane, piperidine and pyridine alkaloid biosynthesis” (72 genes), “glucosinolate biosynthesis” (60 genes), “synthesis and degradation of ketone bodies” (35 genes), “indole alkaloid biosynthesis” (22 genes), “anthocyanin biosynthesis” (20 genes), and “betalain biosynthesis” (4 genes). Plants often produce secondary metabolites for pathogen defense, so it is unsurprising that 2,348 unigenes were mapped to pathways involved in plant-pathogen interactions, such as “phagosomes” (422) and “natural killer cell-mediated cytotoxicity” (126). In addition, many genes were related to “fatty acid biosynthesis and metabolism”; these pathways included “alpha-linolenic acid metabolism” (268), “biosynthesis of unsaturated fatty acids” (216), “fatty acid metabolism” (204), “fatty acid biosynthesis” (143), “linoleic acid metabolism” (105) and “arachidonic acid metabolism” (48). Finally, some genes also distributed to the plant hormone biosynthesis pathways, including “zeatin biosynthesis” (361) and “brassinosteroid biosynthesis” (28).

### Analysis of Flavonoid Biosynthesis-related Pathways and Genes in the Safflower

**Figure 4:**
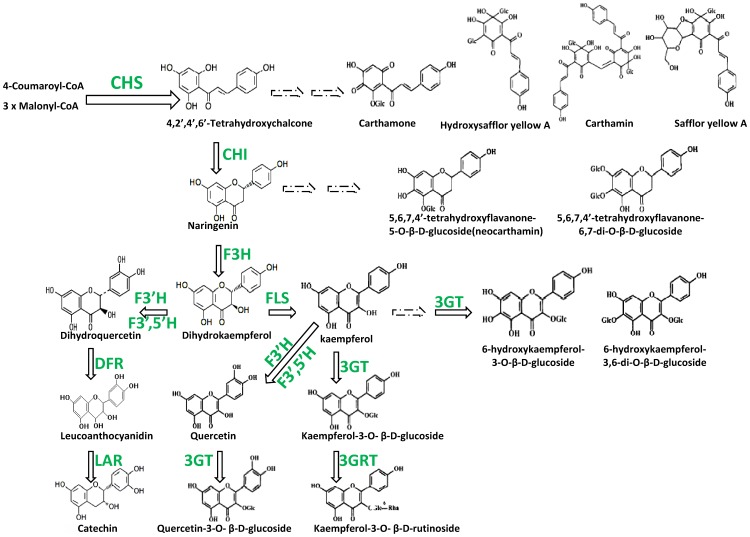
Predicted flavonoid biosynthetic pathway-related genes and products in the safflower flower. The pathways leading to the biosynthesis of different flavonoids. Abbreviations: CHS, chalcone synthase; CHI, chalcone isomerase; F3H, flavanone3-hydroxylase; F3′H, flavonoid 3′-hydroxylase;F3′5′H, flavonoid 3′,5′-hydroxylase; FLS, flavonol synthase; DFR, dihydroflavonol 4-reductase; LAR, leucoanthocyanidin reductase; 3GT, flavonol 3-O-glucosyltransferase; and 3GRT, flavonol-3-O-glucoside L-rhamnosyltransferase. The dotted arrow indicates that the involved enzymes are not yet clear.

The flavonoids include flavanone, flavones, flavonols, quinochalcone and their derivatives. Because the pharmacological effects of safflower flavonoids, we focused on the related genes in this study. In the safflower, quinochalcone is the product of a chalcone, which is oxidized in the A ring and then substituted by glucoses in one or more positions to achieve the final structure. In our annotated safflower flower tissue transcriptome datasets, we identified multiple unigenes encoding enzymes involved in known flavonoid biosynthesis pathways. Their sequencing depth was more than 10-fold coverages (Figure 4, File S2).

The main pathway of flavonoid biosynthesis is well understood, and it is known to be fairly well conserved among plants [30], [31], [32]. At the beginning of this process, one molecule of 4-coumaroyl-CoA and three molecules of Malonyl-CoA are catalyzed by the polyketide synthase, chalcone synthase (CHS, EC 2.3.1.74, in KEGG database), into 4,2′,4′,6′-tetrahydroxychalcone. CHS is the first committed enzyme in this pathway. Five safflower unigenes and two hypothetical unigenes were identified as CHIs; four of them were more than 1000 bp in length. The intermediate, 4,2′,4′,6′-tetrahydroxychalcone, is key for the biosynthesis of both quinochalcones and flavonoids; it may be hydroxylated and glycosylated, leading to the production of carthamone (in red flowers) and hydroxysafflor yellow A. Hydroxysafflor yellow A, which was first identified in 1993 by Meselhy *et al*. [33], is the main component of the red/orange color of their flowers, and it is the most widely studied chemical in the safflower. Previous studies have shown that hydroxysafflor yellow A has the following properties: it can enhance the survival of vascular endothelial cells under hypoxia [34]; it can protect against LPS-induced acute lung injury, which is thought to be associated with the inhibition of p38 MAPK, the activation of NF-kappaB p65, and changes in inflammatory cytokine expression [35]; it might be a promising antifibrotic agent in chronic liver disease [36]; it can significantly inhibit the neuronal damage induced by exposure to glutamate and sodium cyanide (NaCN) in cultured fetal cortical cells [37]; it can protect against mitochondrial injuries in rat cortexes induced by cerebral ischemia [38]; and it can protect human umbilical vein endothelial cells from hypoxia-induced injuries by inhibiting apoptosis and cell cycle arrest [39]. As a substrate, hydroxysafflor yellow A can be catalyzed to carthamin and safflor yellow A. One study showed that injection of safflor yellow A is a safe and effective treatment for coronary heart disease angina pectoris (CHD-AP) with Xin-blood stagnation syndrome (XBSS) [40]. The enzymes responsible for catalyzing 4,2′,4′,6′-tetrahydroxychalcone to quinochalcones are not well understood at this time, so they were not assessed in the present study.

Subsequently, chalcone isomerase (CHI; EC 5.5.1.6) catalyzes 4,2′,4′,6′-tetrahydroxychalcone into naringenin. Four CHI unigenes were identified in the safflower, showing high unique-mapped-reads. In one branch of the biosynthetic pathway in the safflower, naringenin can be hydroxylated at the 6 position and glycosylated at the 5 position to produce 5,6,7,4-tetrahydroxyflavanone-5-O-β -D-glucoside (neocarthamin), or it may be hydroxylated and glycosylated at the 6 and 7 positions to produce 5,6,7,4′-tetrahydroxyflavanone-6,7-di-O-β -D-glucoside. The more highly conserved pathway in plants is for naringenin to be catalyzed by flavanone 3-hydroxylase (F3H, also called naringenin 3-dioxygenase; EC 1.14.11.9) into dihydrokaempferol, which is a very important intermediate product. We identified six naringenin 3-dioxygenases in the safflower.

Dihydrokaempferol can be converted by flavonol synthase (FLS; EC 1.14.11.23) to kaempferol. Eight FLS unigenes were identified in the safflower. Kaempferol can be hydroxylated at its 6 position and then glycosylated by flavonol 3-O-glucosyltransferase (3GT; EC 2.4.1.91) to 6-hydroxykaempferol-3-O- β-D-glucoside or 6-hydroxykaempferol-3,6-di-O-β -D-glucoside. Only one 3GT unigene was identified in the safflower (Unigene7139_safflower; 1,815 bp). Kaempferol can be converted by 3GT to produce kaempferol-3-O- β-D-glucoside, which can then be converted to kaempferol-3-O- β-rutinoside by flavonol-3-O-glucoside L-rhamnosyltransferase (3GRT; EC 2.4.1.159). In our study, kaempferol-3-O- β-rutinoside was commonly found in the white flower tissues. One 3GRT unigene was identified in the safflower flower (Unigene59018_safflower).

Kaempferol can also be converted to quercetin by flavonoid 3′-hydroxylase (F3′H, also called flavonoid 3′-monooxygenase; EC 1.14.13.21) or flavonoid 3′, 5′-hydroxylase (F3′5′H; EC 1.14.13.88) by adding a hydroxyl to the 3′ position of ring B. F3′H was found to be the largest family of safflower flavonoid biosynthesis enzymes, comprising 39 members ranging from 165 bp to 1,456 bp. In contrast, only three F3′5′H unigenes were identified. In the next step of this pathway, 3GT can catalyze quercetin to quercetin-3-O- β-D-glucoside. As an optional pathway, dihydrokaempferol can be converted to dihydroquercetin by F3′5′H or F3′H, and then dihydroquercetin can be deoxidized to leucoanthocyanidin by dihydroflavonol-4-reductase (DFR; EC 1.1.1.219). Three DFR unigenes were identified in the safflower flower. Finally, leucoanthocyanidin can be catalyzed to catechin by leucoanthocyanidin reductase (LAR; EC 1.17.1.3). The LAR family was the second largest family identified in this pathway; 10 members were found in the safflower, ranging from 202 bp to 1,382 bp.

Although we searched for flavone synthase (FNS) genes in the transcriptome sequencing data of the safflower, we did not identify any such genes in this study. We also failed to find any anthocyanidine-encoding genes; however, we did identify four anthocyanidin synthase (ANS, also called leucoanthocyanidin dioxygenase)-encoding genes, suggesting that the genes encoding other anthocyanidine-related products may exist but have not yet been identified in the flower.

### qRT-PCR Analysis of the Flavonoid Synthesis Related Genes in the Safflower

**Figure 5:**
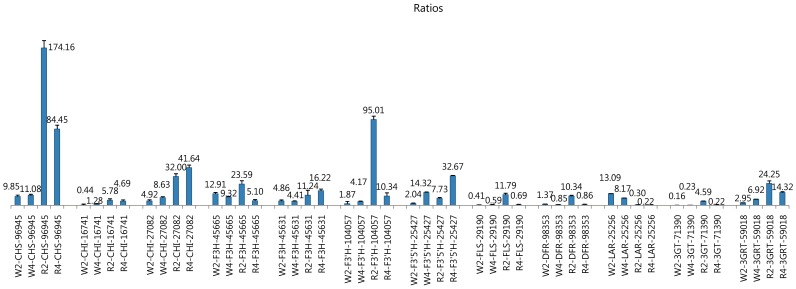
qRT-PCR analysis of 12 flavonoid biosynthetic pathway-related candidate unigenes in white and red flowers. W2, flowering day 2 of the white flower; W4, flowering day 4 of the white flower; R2, flowering day 2 of the red flower; R4, flowering day 4 of the red flower. The numbers after the gene name means the serial number of unigene. The gene sequences used for qRT-PCR analysis are shown in File S5.

To confirm that the unigenes from sequencing and computational analysis were indeed expressed and also to analyze the difference of gene expression profile between red and white color flowers, twelve unigenes related to the ten families of safflower flavonoid synthesis were chosen for qRT-PCR analysis. In the qRT-PCR analysis, to get more information, we used four flower materials including of flowering day2 and day4 of both white and red flowers for further compare instead of day1 and day3 of flowering (Figure 5, the ratios were resulted from the comparing analysis of each gene to 28S, unigene82078).

In general, the red flower showed much higher gene expression profile than the white flower in most of the selected genes. Among them, the CHS gene (unigene96945) showed very high expression in the red flowers with the ratios of 174.16 and 84.5 on flowering day2 and day4 individually; comparing to the ratios in the red flower, this gene showed much lower expression in the white flowers with ratios of 9.85 and 11.08 on flowering day2 and day4 separately. This result indicated that CHS gene (unigene96945) is very important for safflower flavonoid synthesis. The similar expression profile can be observed when considering the two detected CHI genes (unigene16741 and unigene27082); they showed much higher expression in the red flower than in the white flower, especially the unigene27082 with ratios of 32.0 and 41.64. For the two F3H genes (unigene45665 and unigene4561), they showed a relatively medium expression in the two color flowers. The F3′H gene (unigene104057) also showed very high expression on flowering day2 of the red flower with ratio of 95.01, suggesting that at the early stage of flowering (day2), hydroxylation is an important modification for the red flower flavonoid synthesis. Similar expression profiles can also be observed in the genes of FLS (unigene29190), DFR (unigene98353) and 3GT (unigene71390). On the contrary, the F3′5′H gene (unigene2547) showed higher expression in the later stage of flowering (day4) than in the early stage of flowering (day2) in both white and red flowers. Among all of the twelve genes, only the LAR gene (unigene25256) showed much higher expression in the white flowers with ratios of 13.09 and 8.17 on day2 and day4 individually comparing to the ratios of 0.30 and 0.22 on day2 and day4 in the red flowers, indicating that as a substrate much more leucoanthocyandin is produced in the white flower than in the red flower.

## Discussion

High-throughput mRNA sequencing technology is especially suitable for gene expression profiling in non-model organisms that lack genomic sequence data. Prior to this study, most sequencing efforts in the safflower were based on EST sequencing; very few tags had been reported in public databases and little genetic or genomic information was available. Here, we used RNA-Seq technology to profile the safflower flower transcriptome on the Illumina HiSeq™ 2000 platform, obtained 4.6 Gb of coverage with 52,119,104 clean sequencing reads, and identified a total of 120,778 unigenes from *de novo* assembly. Among them, 70,342 unigenes were successfully annotated (about 58% of the assembled unigenes), suggesting their relatively conserved functions. We assessed not only the gene or protein names and descriptions, but also their putative conserved domains, gene ontology terms, and potential metabolic pathways. This work will certainly improve our understanding of the processes involved in regulating the biosynthesis of secondary metabolites and the development of safflower flower tissues. Compared with previous transcriptomic studies in other plants, such as *Acacia auriculiformis*, *Acacia mangium* [41], *Eucalyptus* [42], and *Taxus* [43], we herein report more contigs and unigenes, suggesting that the safflower contains very abundant gene resources. To the best of our knowledge, this is the first attempt using Illumina paired-end sequencing technology for the *de novo* sequencing and assembly (without a reference genome) of the safflower flower transcriptome. We believe our data will provide important new insights and facilitate further studies of safflower genes and their functions.

In terms of medicinal and pharmaceutical use, the properties of the safflower flower largely depend on its flavonoid profile. Since gaining new insight into the biosynthesis and transcriptional regulation of flavonoids in the safflower should accelerate the engineering of this pathway in the future, we focused on the flavonoid biosynthesis genes identified in the present work. Almost all of the genes for the main flavonoid biosynthesis pathway were identified (except for the flavone synthesis gene), indicating that this pathway was rather well conserved in the safflower (Figure 4 and File S3). Many of these genes appeared to form multi-gene families, implying that the genome of the safflower, like those of many other higher plants, went through one or more rounds of genome duplication during its evolution. However, the actual flavonoid pigments of plants are highly diverse in structure and color, reflecting the diversity of plants and their metabolic pathways. Indeed, the safflower is likely to possess some unique, yet-unidentified flavonoid-related molecules. Therefore, we further surveyed other genes that might be involved in the various branch or terminal pathways of flavonoid biosynthesis. KEGG predictions allowed us to identify an additional 227 genes that also contributed to generalized flavonoid biosynthesis, including members of the CYP family (electron carrier/heme binding/iron ion binding/monooxygenase/oxygen binding), the O-methyltransferase, O-hydroxycinnamoyltransferase family, and the glycosyltransferase family, which are involved in uniquely modifying the flavonoid skeleton to yield the final products (the other related genes are given in File S3).

The safflower is a multipurpose crop, but its main use worldwide is as an oil crop. Its main seed oil components are linoleic acid (71–75%), oleic acid (16–20%), palmitic acid (6–8%) and stearic acid (2–3%) [1], most of which are unsaturated fatty acids. Safflower petals also contain alpha linolenic acid (15–19%), palmitic acids (14–16%), gamma linolenic acid (2–3%), and decanoic and dodecanoic acids (2–5%) [44]. Safflower linoleic acid may be beneficial for weight loss and/or glycemic control for type-2 diabetes [45], while dietary supplementation with safflower oil and olive oil was shown to rescue aberrant embryonic arachidonic acid and nitric oxide metabolism and prevent embryopathy in diabetic rats [46]. In our sequencing of transcripts from 1–3d flowers, we identified 149 genes that distributed to the “biosynthesis of unsaturated fatty acids” category (File S4; sequencing depth >10). These were mainly fatty acid desaturases, including stearoyl-ACP desaturase, which was cloned in 1992 by Knutzon *et al*. [47], along with FAD2, FAD3, FAD5A, FAD6, FAD7 and FAD8 [48], [49], [50]. The expression of these gene products during this flowering stage suggests that the unsaturated fatty acid biosynthesis pathway is rather well conserved in the safflower with respect to Arabidopsis, Rape and Cabbage. Although we only identified one oil biosynthesis gene in safflower flowers, namely that encoding stearoyl-ACP desaturase, our data may facilitate the future study of other related genes.

In conclusion, this sequence collection represents the first major genomic resource for the safflower, *Carthamus tinctorius* L. Using Illumina sequencing technology, we surveyed the flower transcriptome of the safflower, assembled 120,778 unigenes and annotated 70,342 of these unigenes. These findings provided comprehensive coverage, suggesting that we identified almost of the all known genes from the major metabolic pathways operating in the safflower flower. Our KEGG prediction results suggest that the flavonoid biosynthesis and unsaturated fatty acid biosynthesis pathway-related genes are rather well conserved in the safflower. Overall, we identified more than 200 pathways as being represented in the safflower whole-flower transcriptome. Further analysis of these pathway-related genes will improve our understanding of the safflower’s unique and common features. This study again demonstrates that Illumina sequencing technology may be applied as a rapid and cost-effective method for *de novo* transcriptome analysis of non-model plants for which genomic information is unavailable. We believe that this transcriptome dataset will serve as an important public information platform to accelerate research on the gene expression, genomics, and functional genomics of *Carthamus tinctorius* L.

## Materials and Methods

### Tissue Collection and RNA Isolation

Safflower plants were cultivated in the traditional Chinese medicine cultivation zone of the town of Shipan (Jianyang county Sichuan Province, China; 30.24?N 104.32?E). This zone was built by our institute (Institute of Economic Crops Breeding and Cultivation, Sichuan Academy of Agricultural Sciences) for the collection and cultivation of Chinese herbal medicine resources. The safflower is not on the list of endangered/protected plants, and no specific permission was required for us to use this material. The safflower is generally planted in late October, and then flowers during the first two weeks of the following May. Tubular flower tissues of 1-day-old (1d), 2d and 3d were cultivated from red/orange and white *Carthamus tinctorius* L. The white flower is a normally occurring mutant of the red/orange flower (Figure 1A). Total RNA was isolated from each sample using TRIzol (Invitrogen) according to the manufacturer’s instructions. Total RNA was treated with RNase-free DNase I (New England BioLabs) for 30 min at 37°C to remove residual DNA. Equal amounts of RNA from each sample were mixed for the subsequent steps of our experiments. The workflow is shown in Figure 1B.

### Preparation and Sequencing (mRNA-seq) of the cDNA Library

The workflow described in this section is shown in Figure 1B. In brief, RNA was collected from flower tissues, and oligo(dT) beads were used to isolate poly(A) mRNA. Fragmentation buffer was used to chop the mRNA into short fragments, which were then used as templates for random hexamer-primed synthesis of first-strand cDNA. Second-strand cDNA was synthesized using buffer, dNTPs, RNase H, and DNA polymerase I. From these cDNA, a paired-end library was synthesized using the Genomic Sample Prep kit (Illumina), according to the manufacturer’s instructions. Short fragments were purified with the QIAquick PCR (Qiagen) extraction kit and then resolved with EB buffer for end repair and the addition of poly (A). The short fragments were then connected with sequencing adapters, and suitable fragments were separated by agarose gel electrophoresis. Finally, the sequencing library was built by PCR amplification and sequenced using the HiSeq™ 2000 platform (Illumina). The transcriptome datasets are available at the NCBI Sequence Read Archive (SRA), under accession number SRA048496.

### Analysis of Illumina Transcriptome Sequencing Results

A simplified workflow of the transcriptome assembly and bioinformatic analysis is shown in Figure 1C and 1D, respectively. The raw sequencing data were transformed by base calling into sequence data (called raw data or raw reads), which were stored in fastq format. Adaptor fragments were removed from the raw reads to yield the clean reads required for analysis. *De novo* transcriptome assembly of these short reads was performed using the SOAPdenovo assembling program, which first combined reads with a certain length of overlap to form longer fragments without N-called contigs. The reads were mapped back to contigs, and paired-end reads were used to detect contigs arising from the same transcript as well as the distances between these contigs. Next, SOAPdenovo connected the contigs, using “N” to represent unknown sequences, yielding scaffolds. Paired-end reads were then used again to fill in the gaps between the scaffolds, yielding sequences that had the fewest Ns and could not be extended on either end. These were designated as unigenes.

For further analysis, we first used BLASTX (E-value <10^−5^) to search the unigene sequences against various protein databases, in the following order: Nr, SwissProt, KEGG and COG. Unigene sequences having hits in one database were not used to search the subsequent database(s). The BLAST results were then used to extract CDS from the unigene sequences, and translate them into peptide sequences. The BLAST results were also used to train ESTScan. The CDS of unigenes that had no BLAST hits were predicted by ESTScan and then translated into peptide sequences. Unigene annotation provided information on the expression patterns and functional annotations of the identified genes. For Nr annotation, we used the BLAST2GO program to obtain the GO annotations of the unigenes, and then used the WEGO software to perform GO functional classifications for all the unigenes and to explore the macro-distribution of gene functions for this species.

### Analysis of Metabolic Pathway Genes Identified from Carthamus Tinctorius L. Flowers

The metabolic pathway analysis was done using the Kyoto Encyclopedia of Genes and Genomes (KEGG) database and related software applications (http://www.genome.jp/kegg/kegg4.html), which form a bioinformatics resource for linking genomes to living organisms [51]. Within the KEGG databases, the PATHWAY database contains information on networks of molecular interactions within cells, as well as the variations specific to particular organisms. The genes involved in flavonoid and unsaturated fatty acid biosynthesis were identified using BLAST annotation of KEGG and the other databases mentioned above. To increase the credibility of our results, we selected genes that had coverages >10.

### Gene Validation and Expression Analysis

Twelve selected unigenes with potential roles in flavonoid synthesis were chosen for validation using real time qPCR with gene specific primers designed with primer premier 3.0 (the sequences of the selected genes was shown in File S4 and the primers designed for qRT-PCR analysis was shown in File S6). Total RNA was extracted from day2 and day4 of red and white flowers individiually using trizol RNA extraction method (Invitrogen, USA) and purified with RNA purification kit (Qiagen, USA). The purified RNA was reverse transcript to cDNA using PrimeScrit® RT reagent kit with gDNA Eraser (Perfect Real Time) (Takara, China). 14 genes were first chose for RT-PCR analysis and 12 of them were further chose for qRT-PCR. All the genes used for qRT-PCR were first performed RT-PCR analysis and run gel to get a good condition for qRT-PCR analysis. The qRT-PCR reaction was performed according to the protocol of Green Mastermix (Qiagen, USA) using ABI 7500 system. Three biology duplications of each sample (each sample containing flowers from 4 individual plants) and triplicates of each reaction were performed. 28S gene was chosen as an internal control for normalization after comparison the expressions of two reference genes (15S, unigene104038 and 28S) in different materials.

## Supporting Information

File S1 **Summary of the involved KEGG pathways and their corresponding gene numbers.**
(XLSX)

File S2 **The main identified flavonoid biosynthetic pathway genes and their coverage.**
(XLSX)

File S3 **The other flavonoid biosynthetic pathway-related genes and their coverages.**
(XLSX)

File S4 **Genes of the safflower flower transcriptome identified as contributing to “biosynthesis of unsaturated fatty acids.”**
(XLSX)

File S5 **The gene sequences used for qRT-PCR analysis.**
(TXT)

File S6 **The primers designed for qRT-PCR analysis.**
(XLS)
